# 5-ALA fluorescence in indeterminate grade gliomas

**DOI:** 10.3171/2021.10.FOCVID21196

**Published:** 2022-01-01

**Authors:** Michael Müther, Walter Stummer

**Affiliations:** Department of Neurosurgery, University Hospital Münster, Germany

**Keywords:** 5-ALA, aminolevulinic acid, fluorescence-guided surgery, low-grade glioma

## Abstract

5-Aminolevulinic acid (5-ALA) is a useful and well-established adjunct for glioblastoma surgery. A growing body of evidence has revealed the potential utility of 5-ALA in grade II and grade III glioma patients as well. However, reliable means of identifying in whom fluorescence will occur have not been established. The authors report the case of such an indeterminate-grade glioma highlighting two pearls of 5-ALA fluorescence in this subgroup of patients. Firstly, 5-ALA–guided tissue sampling helps to ensure that the true grade of the lesion is not underestimated. Secondly, intraoperative fluorescence can serve as a prognostic marker.

The video can be found here: https://stream.cadmore.media/r10.3171/2021.10.FOCVID21196

## Transcript

Here, we present the case of a 37-year-old otherwise healthy female who experienced progressive headaches for several months. A subsequent MRI demonstrates a left frontal lobe space-occupying lesion. The lesion is hyperintense on T2 with a T2/FLAIR signal mismatch. There is focal contrast enhancement over the center of the lesion, as shown on the right image. For further evaluation, an FET-PET was obtained. An obvious hypermetabolic hot spot can be appreciated toward the cortical portion of the lesion. In addition, a second hypermetabolic spot can be seen around the area of MRI contrast enhancement.

### 1:10

Given the T2/FLAIR mismatch, IDH mutation was probable. Still, according to the high metabolic activity as provided by the FET-PET, the grading of the lesion was unclear. The patient consented for microsurgical fluorescence-guided resection. We anticipated a risk to speech- and language-associated subcortical structures. Therefore, the procedure was conducted in a standard awake setting.

### 1:34 Intraoperative Fluorescence.

Starting with defining the posterior border of the tumor, intraoperative fluorescence was detected early. The factors associated with visible fluorescence in these low- and intermediate-grade gliomas have not been fully determined. According to a recent systematic review, however, factors such as proliferative activity, cellular density, blood-brain barrier permeability, vascularity, and anaplasia may correlate with the intensity of low-grade glioma fluorescence.^1^

### 1:54

Generally, visible fluorescence is observed in 98%–100% of glioblastoma patients treated with 5-ALA.^2,3^ In contrast, visible fluorescence is observed in approximately 75%–85% of all grade III gliomas and 16%–20% of grade II diffuse gliomas.^2,4^ In those cases, 5-ALA fluorescence is reported to correlate with signal intensity on FET-PET.^2,5,6^ Here, fluorescence was coregistered with the available preoperative imaging data using neuronavigation. The location of fluorescing tissue exactly matched the minor spot of FET-PET positivity over the area of focal contrast enhancement. Separate biopsies were taken from both areas of FET-PET positivity including the fluorescing part of the tumor to best represent intratumoral heterogeneity. This helps not to underestimate the true grade of the lesion.^5^ Especially due to shift in tissue masses during resection of large lesions, finding an anaplastic focus can be challenging.

### 2:50 Gross-Total Resection.

Gross-total resection was achieved, and the further postoperative course was uneventful. After histopathological and molecular analyses, the diagnosis of IDH1 (R132C)–mutated diffuse astrocytoma (grade II WHO) was established. ATRX expression was lost, CDKN2A/B was retained, and the tumor was of increased cellular density with an MIB-1 of 13%. The patient exhibited various favorable prognostic factors: age < 40 years, no neurological deficit, low tumor burden, and grade II pathology. Also, the patient was reluctant concerning adjuvant therapies. In line with current practice management guidelines, a decision was made to watch and wait.^7^

### 3:16 Follow-Up MRI.

Early in the course of disease, 1 year after initial gross-total resection, follow-up MRI revealed tumor progression and the patient underwent a reresection. Again, tissue fluorescence was encountered. The diagnosis again was IDH-mutated diffuse astrocytoma. This was further confirmed by additional DNA methylation analysis. Now, decision was made to start sequential radiochemotherapy. The patient has been stable for 2 years now.

### 3:59 Case Highlights.

This case highlights two important aspects that support the use of 5-ALA in indeterminate-grade gliomas. Firstly, 5-ALA–guided tissue sampling helps not to underestimate the true grade of the lesion. Secondly, 5-ALA fluorescence was found to be an unfavorable prognostic marker in low-grade glioma patients. This finding was first published by our group in 2019 and recently corroborated by a transatlantic initiative.^4,8^ This translates into a potential for 5-ALA fluorescence to unveil high-risk, low-grade patients. The molecular substrate underlying this phenomenon is currently under investigation.

According to current clinical management guidelines, for certain cases of IDH-mutant diffuse astrocytoma, robust factors that support adjuvant treatment over a watch-and-wait strategy are lacking.^7^

Coming back to the case presented, with the data on prognostic impact of 5-ALA fluorescence now available, the notion of intraoperative fluorescence might have led to a stronger recommendation toward adjuvant treatment in the first place. With this, early tumor progression might have been prevented. However, the influence of fluorescence on decision-making processes and resulting outcome will be within the scope of future studies.

Finally, with advancements in visualization techniques and administration schemes of 5-ALA, fluorescence-guided resections comparable to surgery in high-grade gliomas may be feasible soon.

## Disclosures

Dr. Stummer reported personal fees from NXDC, SBI ALAPharma, and Medac during the conduct of the study.

## Author Contributions

Primary surgeon: Stummer. Assistant surgeon: Müther. Editing and drafting the video and abstract: both authors. Critically revising the work: both authors. Reviewed submitted version of the work: both authors. Approved the final version of the work on behalf of all authors: Müther. Supervision: Stummer.
